# A Promising New Strategy to Improve Treatment Outcomes for Patients with Depression

**DOI:** 10.1089/pop.2018.0101

**Published:** 2019-05-30

**Authors:** George Carpenter, Henry T. Harbin, Robin L. Smith, John Hornberger, David B. Nash

**Affiliations:** ^1^ MYnd Analytics, Mission Viejo, California.; ^2^ Health Care Consultant, Baltimore, Maryland.; ^3^ Stanford University, Stanford, California.; ^4^ Cedar Associates, Menlo Park, California.; ^5^ Jefferson College of Population Health, Philadelphia, Pennsylvania.

**Keywords:** behavioral health, predictive modeling, clinical testing

## Abstract

Each year, ineffective medical management of patients with mental illness compromises the health and well-being of individuals, and also impacts communities and our society. A variety of interrelated factors have impeded the health system's ability to treat patients with behavior health conditions adequately. A key contributing factor is a lack of objective markers to help predict patient response to specific drugs that has led to patterns of “trial and error” prescribing. For many years, clinicians have sought objective data (eg, a laboratory or imaging test) to assist them in selecting appropriate treatments for individual patients. Electroencephalogram (EEG) findings coupled with medication outcomes data may provide a solution. “Crowdsourced” physician registries that reference clinical outcomes to individual patient physiology have been used successfully for cancers. These techniques are now being explored in the context of behavioral health care. The Psychiatric EEG Evaluation Registry (PEER) is one such approach. PEER is a clinical phenotypic database comprising more than 11,000 baseline EEGs and more than 39,000 outcomes of medication treatment for a variety of mental health diagnoses. Collective findings from 45 studies (3130 patients) provide compelling evidence for PEER as a relatively simple, inexpensive predictor of likely patient response to specific antidepressants and likely treatment-related side effects (including suicidal ideation).

## Introduction

Despite unprecedented progress in understanding and treating *physical* illness, effective medical management of patients with *mental* health conditions remains among the most daunting and complex population health issues in the United States today. National population statistics paint a bleak picture of the burden of mental illness on the population.

According to a nationwide survey, an estimated 4.0% (9.8 million) of all US adults (aged 18 years or older) experienced a serious mental illness, and an astounding 17.9% (43.4 million) of all US adults experienced any mental illness at some points in their lives.^1^ The same study reported that 16.1 million adults (an estimated 6.7% of the total adult population) had at least 1 major depressive episode in the previous year.^1^

The broad impact for individuals with depression, their families, and society in general – especially during the early onset of the condition – include reduced educational attainment, increased risk of teen childbearing, marital disruption, and unstable employment. Major depressive disorder (MDD) also has been associated with a wide range of chronic physical disorders and early mortality.^2^ A 2015 study from Greenberg and colleagues estimated that nearly half of the total $210.5 billion economic burden of MDD is attributable to workplace issues such as absenteeism and presenteeism (reduced productivity while at work).^3^

Although the level of national funding for behavioral health care has increased as a result of targeted legislation (eg, the Patient Protection and Affordable Care Act [2010], the Mental Health Parity and Addiction Equity Act [2008]), questions have arisen regarding the value of current guidance/tools and standard psychiatric treatment options that generate little or no incremental impact on population health.

Multiple interrelated challenges hamper the health care system's ability to address these issues, including:

### A shortage of psychiatrists

The chronic shortage of psychiatrists – particularly in poorer urban and rural areas – has long been a barrier to Americans who need mental health care services. As a consequence of the shortage, it often falls to nonpsychiatrist physicians (eg, primary care providers) to treat patients with mental health conditions. Although it is unclear whether primary care practitioners are well equipped to manage depression as a chronic illness, more than half of the 8 million ambulatory care visits for depression each year are to a primary care physician.^4,5^

Regardless of physician specialty, epidemiologic research shows that, although mental health disorders affect tens of millions of Americans each year, only half of those with symptoms actually seek and/or receive treatment – and the treatment provided is ineffective for a majority of those who receive it. For example, of patients treated for a mental health condition by their primary care provider, only 12.7% receive “minimally adequate treatment.”^6^

### Ineffectiveness of commonly used drugs and prescribing patterns

The ineffectiveness of prescribed medications and their related side effects are associated with high rates of nonadherence and medical treatment dropout.^7^ Some commonly prescribed medications must be taken for 4–6 weeks before having a measurable effect. Typical side effects include weight gain, diminished libido, and diminished sexual function.

Most important, individual patients vary widely and unpredictably in their response to specific medical treatments. Many patients who are not helped by first-line treatments – particularly those treated in community primary care practices – are not offered an alternative treatment.^8^ Clearly, the ability to accurately select the initial treatment would be beneficial.

### Lack of objective markers for treatment of mental health conditions

Perhaps the most serious and consequential concern lies in the relative lack of objective, evidence-based predictive markers to inform pharmacologic treatment for mental health conditions. Current psychiatric practice patterns – often described as “trial and error pharmacotherapy” – result in millions of patients being labeled “resistant to treatment” after failing to respond to 2 or more drugs, often from the same therapeutic class.^9^

In addition to subjecting patients to ineffective treatment and/or undesired side effects, the estimated cost of “trial and error” to payers is significant. In a recent study of treatment-resistant depression, patients who failed 2 or more treatments had costs that were roughly double ($17,261 per year) those of standard depression patients ($9790) and quadruple compared with people without depression ($4782). These costs are attributable to an almost 2-fold increase in the number of office visits and more than 3 times the number of inpatient claims compared with patients who are not “treatment resistant.”^10^

## Breakthrough in Decision Support for Mental Health Providers

Clinical tests, measures, and evidence-based guidance are available in abundance for physicians who treat patients for acute and chronic physical conditions; contrast the US health system's well-defined, deliberate approach to diabetes management with its relatively casual approach to depression. The disparity is especially evident in quality measurement and reporting requirements. For example, the widely used Healthcare Effectiveness Data and Information Set (HEDIS) – a performance measurement tool with 94 quality measures across 7 domains of patient care – includes only 1 measure for depression. Ironically, the chronic conditions that are the focus of many HEDIS measures (eg, diabetes, cardiac disease) frequently occur as comorbid conditions with depression.

The electroencephalogram (EEG), a test that uses an electronic monitoring device to measure and record electrical activity in the brain, has long been a vital tool for diagnosing and managing seizure disorders and in evaluating brain damage, mental retardation, degenerative diseases (eg, Alzheimer's disease), and certain mental health disorders (eg, substance dependence, schizophrenia, autism). EEG studies are used to reference large normative populations with baseline EEGs and subsequent tracking of treatment interventions and outcomes.

Today, researchers are seeking objective tests and measures to help identify comparably safe and effective therapies to minimize uncertainty when treating patients with mental illness. A promising new strategy combines a standardized, well-normed, and ubiquitous technology (EEG) with a clinical registry of reported outcomes for patients receiving pharmacotherapy.

### Brief history of quantitative EEG recordings

Beginning in the 1970s, EEG recordings have been digitized and subjected to quantitative analysis (QEEG) to provide physicians with more detailed information than is available from a visual inspection. Statistically significant variations found in QEEG patterns within various neuropsychiatric disorders enabled early researchers to define clinically meaningful subgroups based on QEEG findings.^11^

Subsequent findings from studies by Leuchter,^12^ Arns,^13–15^ Pizzagalli,^16^ and others confirmed that patients within the same neuropsychiatric disorder might be characterized by their propensity to respond to certain medications (and not others) and that these propensities could be revealed by QEEG. These findings were replicated in the recently reported EMBARC (Establishing Moderators and Bio-signatures of Antidepressant Response for Clinical Care) trial at 4 US sites in which more than 300 patients with MDD were evaluated through brain imaging and various DNA, blood, and other tests. As the study of brain imaging and blood biomarkers continues, EMBARC trial organizer, Madhukar Trivedi, MD, recommended that patients request these tests when seeking an antidepressant.^12^

Following a strategy that was successful in dramatically reducing childhood cancers, the next advance in the field involved development of a large QEEG outcomes registry that could accurately correlate individual QEEG signatures with patients who had effective medication response.

Large “crowdsourced” physician registries that reference clinical outcomes to individual patient physiology have been used successfully in managing pediatric cancers. Such population health techniques can be applied to behavioral health care. Mynd Analytics – one of several organizations conducting research in this sphere – has designed, clinically tested, and achieved significantly improved outcomes using a QEEG-based personalized mental health strategy.

### The Psychiatric EEG Evaluation Registry (PEER)

**FIG. 1.**
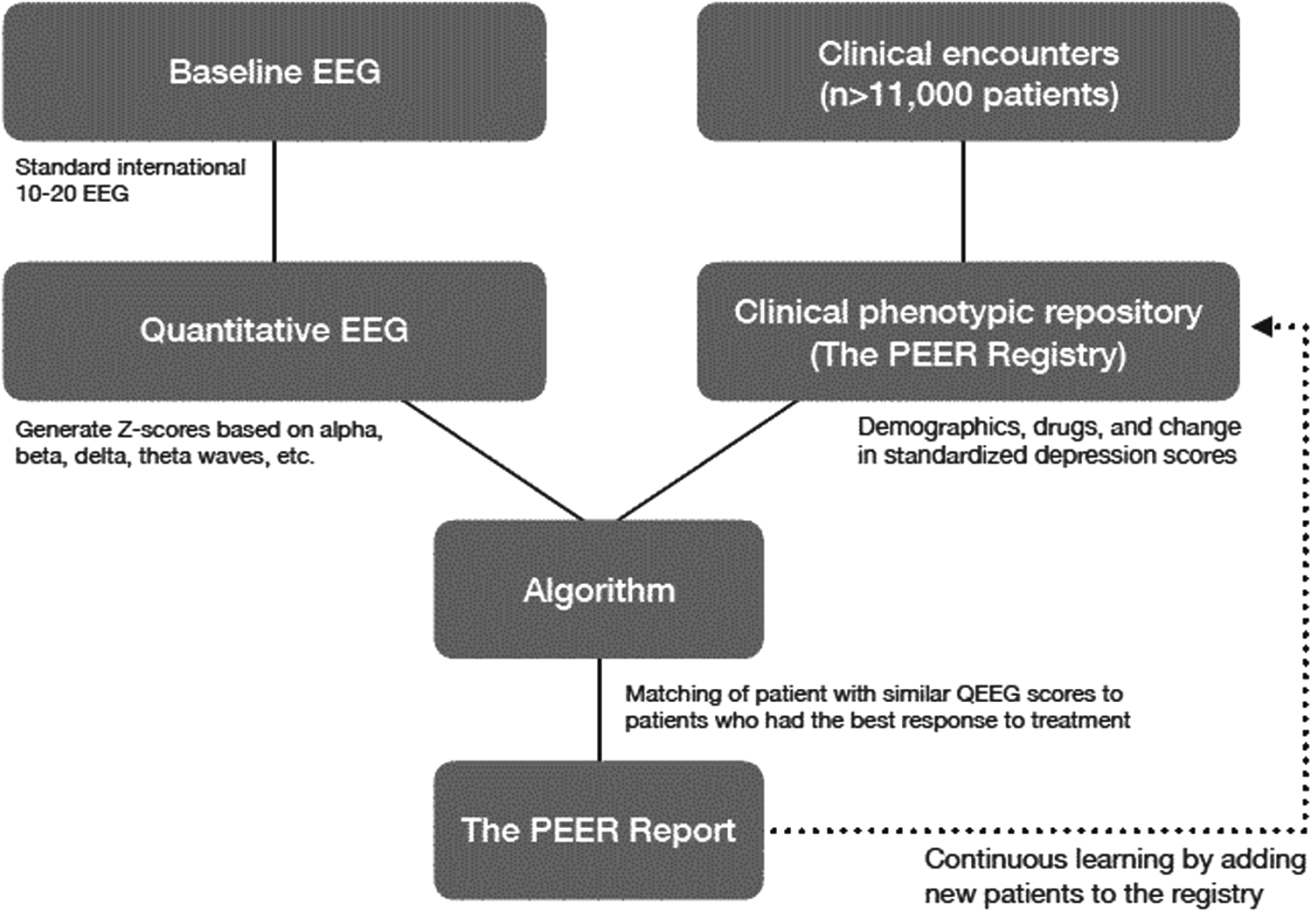
The Psychiatric EEG Evaluation Registry (PEER) process. EEG, electroencephalogram; QEEG, quantitative EEG.

A clinical phenotypic repository, the PEER database comprises more than 11,000 baseline EEGs and more than 39,000 outcomes of medication treatment for a variety of mental health diagnoses (Fig. 1). When applied to large, feature-rich data sets such as results of neuroimaging studies, machine learning approaches can help determine predictive features that explain variation in medication response for individual patients. Features representing variables that may be predictive include clinical, demographic, social, physiological, cognitive, neural, or genetic information. Machine learning algorithms are trained and tested repeatedly using multiple cross-validation techniques (eg, k-fold cross validation), optimizing metrics such as binary responder/nonresponder status based on a threshold of reduction in symptoms. Best practice models — those that perform well on both unseen internal and external data sets — are typically deployed in randomized controlled trials in which treatment allocation based on accepted clinical guidelines is compared to prediction-guided treatment assignment.^17^

The Registry links automated, quantitative EEG findings with phenotypes (ie, drug response in patients with treatment-resistant depression) and correlates long-term therapeutic outcomes for patients with neuroimaging reference data in the form of QEEG. The Registry has driven the development of a series of online tools, including Referenced-EEG, PEER Online, and PEER Interactive, all of which are the functional equivalent of PEER.

The PEER process begins with the treating physician (a psychiatrist or primary care physician) and the patient agreeing to an EEG test to measure the patient's unique brain patterns. This widely available, noninvasive test is inexpensive and generally reimbursed by payers. The patient's EEG is compared to the PEER database, and a proprietary algorithm is used to generate a report for the treating physician within 24 hours. In addition to presenting the relative likelihood of the individual patient's response to each medication class and specific drugs within each class, the report provides additive insight regarding the classes of drugs that are most – and least – likely to produce a favorable outcome for a patient with treatment-resistant depressive disorder.

With a rapidly growing evidence base (12 randomized controlled trials of QEEG neurometrics for predicting medication response and 88 observational cohort studies), PEER shows promise as a tool to assist physicians in selecting the treatment options that are most likely to be effective for each patient. The value proposition for population health lies in improved outcomes for patients with mental health conditions, coupled with reduced direct and indirect costs of mental health care.

## Randomized Controlled Trial (RCT) Outcomes

Collective findings from 45 studies (3130 patients) reported by Wade and Iosifescu^11^ provided compelling, independent evidence for QEEG as a relatively simple, inexpensive predictor of likely patient response to specific antidepressants and likely treatment-related side effects (including suicidal ideation). In particular, the following 4 RCTs of PEER found that patients receiving PEER-guided pharmacotherapy exhibited significantly greater improvement in the severity of depression and in functional outcomes.

1. In 2007, Suffin et al conducted a small RCT that compared outcomes in chronic refractory MDD with and without medication prescribing guided by QEEG. The pretreatment EEG data accurately predicted medication response in 6 of 7 patients compared with only 1 in 6 patients in the group treated without EEG guidance.^18^
2. In 2009, DeBattista et al conducted a multicenter RCT to compare the outcomes of treatment based on a commonly used guidance tool (the Texas Medication Algorithm Project [TMAP]) with the outcomes of an intervention group of 18 patients whose treatment was based on QEEG-guided options. All study patients had failed at least 3 prior antidepressant regimens and all had undergone a washout of current medications. Results at 10 weeks showed that patients whose treatments were guided with QEEG had significantly better outcomes than those medicated according to the TMAP standard in terms of significantly improved Quick Inventory of Depressive Symptomatology (QIDS) and Quality of Life (QoL) scores.^19^
3. Two years later, DeBattista et al conducted a similar 12-center RCT comparing QEEG-guided pharmacotherapy with treatment guided by the most effective regimens reported in the National Institutes of Health-sponsored Sequenced Treatment Alternatives to Relieve Depression Study. Subjects (114) were relatively resistant to treatment (ie, failed on 1 or more antidepressants) and underwent a washout of all current medications. At 12 weeks, results revealed that the QEEG-guided pharmacotherapy group (57 patients) was associated with significantly greater improvement than the control group (57 patients) for 2 primary end points: QIDS-Self Report 16 (−6.8 vs. −4.5, *P* < 0.0002) and QoL Enjoyment and Satisfaction Questionnaire-Short Form (18.0 vs. 8.9, *P* < 0.0002), as well as showing statistical superiority in 9 of 12 secondary end points.^20^
4. Set in 2 military hospitals, a recent RCT conducted by Iosifescu et al (2016) compared PEER-informed pharmacotherapy with Veterans Administration/Department of Defense guidelines/current standard of care in treating participants with a primary *Diagnostic and Statistical Manual of Mental Disorders, 4^th^ edition* diagnosis of depressive disorder. Eligible patients were randomly assigned to intervention and control groups, and analysis was conducted on patients who remained in the study at 6 months. QIDS-SR 16 depression scores were the primary efficacy end point. An evaluation of the predictive validity of PEER recommendations found greater improvements in depression scores (QIDS-SR 16, *P* < 0.03), a reduction in suicidal ideation (Concise Health Risk Tracking Scale – SR 7, *P* < 0.002), and post-traumatic stress disorder score improvement (PTSD Checklist Military/Civilian, *P* < 0.04) for participants treated with PEER-recommended medications versus patients whose physicians did not follow PEER recommendations.^21^

**FIG. 2.**
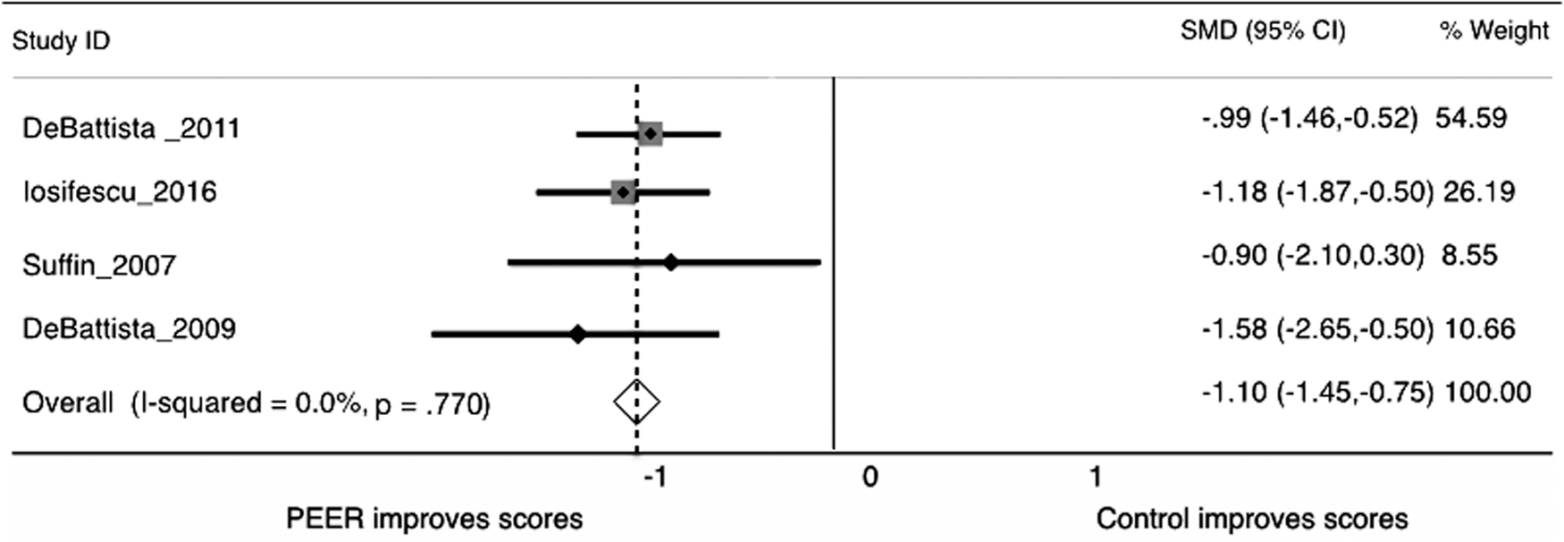
Forest plot of the standard mean difference across the 4 PEER randomized trials versus control treatments (eg, TMAP, STAR*D guided protocols). PEER, Psychiatric Electoenceophalogram Evaluation Registry; STAR*D, Sequenced Treatment Alternatives to Relieve Depression; TMAP, Texas Medication Algorithm Project.

A meta-analysis of these 4 RCTs yielded strong evidence for PEER-guided pharmacotherapy as a targeted strategy for treating depression (Hornberger J; unpublished data; 2017) (Fig. 2). The PEER strategy was associated with a strong positive effect for symptom scales when compared with a moderate or weak effect for current/usual treatment guidelines. Researchers concluded that the patients of physicians who followed recommendations of the PEER Report had 144% greater improvement in depression scores and 75% greater reduction in suicidality when compared with current standard of care treatment. Moreover, treatment that followed PEER recommendations resulted in 2.5 times greater adherence to therapy, defined as continued participation over a 6-month course of treatment.

PEER provides outcome information on more than 90% of the most frequently prescribed medication classes and agents, including the most frequently used antidepressant, atypical antipsychotic, benzodiazepine, stimulant, anticonvulsant, and mood stabilizer agents. The database includes outcomes from 11,000 commercial tests and is updated with new outcomes on a regular basis.

These findings have particular significance in treating patients with depression. In the selective serotonin reuptake inhibitor (SSRI) antidepressant medication class, the average Number Needed to Treat (NNT) is 7; that is, 6 patients will be treated unsuccessfully for every 1 patient who achieves a meaningful clinical response. Physicians using PEER guidance reduced the occurrence of trial-and-error and achieved an NNT of 5, reflecting both greater efficacy and improved patient adherence. Results of NNT analyses mirror results of claim-based budget impact models, which show a 4.7 to 1 net cost offset when tools such as PEER are applied to reduce trial and error treatment (Hornberger J; unpublished data; 2017).

## Discussion

A strategy employing a “crowdsourced” registry aligns well with the principles of population health. Large registries such as PEER have potential when used to obtain longitudinal, durable, therapeutic outcomes in a real-world environment.

The demonstrated superiority of the PEER strategy over a range of current/usual treatment guidelines represents an opportunity to improve the health outcomes of patients with mental health conditions. As each patient's data are entered, the PEER database's accuracy improves via machine learning.

In addition to improving the efficiency and efficacy of pharmacotherapy for patients with depression, the relatively inexpensive, personalized PEER approach may prove to be helpful in addressing a critical challenge: improving access to mental health services by increasing the accuracy of therapeutic decisions and facilitating treatment planning – especially in areas with a shortage of psychiatrists.

### Integrated care management

Primary care providers have increasingly integrated care management processes for chronic physical conditions (eg, diabetes) into their practices. Although more than half of the 8 million ambulatory care visits for depression each year are to a primary care physician, studies reveal that care management processes are used less often for depression than for any other chronic conditions in US primary care practices.^22^ The PEER strategy provides well-evidenced prescribing guidance for managing depression in the primary care setting.

### Collaborative care models (CCM)

CCMs provide the structure for delivering integrated mental health and general medical care in primary care settings.^23^ Team-based, multicomponent interventions, CCMs improve coordination of patient care through evidence-based provider decision making, clinical information systems, and patient engagement. Recent systematic reviews show CCMs to be a cost-efficient strategy for primary care practices to improve mental and physical outcomes for a range of mental health conditions across diverse populations and primary care settings.^24,25^ Used as a tool in the CCM setting, the PEER strategy holds potential to improve the efficacy and efficiency of treatment for depression.^26^

### Telepsychiatry

The term telepsychiatry describes the delivery of psychiatric assessment and care through telecommunications technology (eg, videoconferencing). In areas with shortages of psychiatrists, PEER could be useful as a tool to assist primary care physicians in combination with telepsychiatry.

## Conclusion

Ineffective medical management of patients with mental health conditions – particularly in treating patients with depression – remains a serious population health issue as evidenced by the 25% increase in suicide rates over the past decade. The lack of reliable predictive markers to guide pharmacologic treatments has led to ineffective “trial and error” prescribing. “Crowdsourced” physician registries that reference clinical outcomes to individual patient physiology measured by EEG have demonstrated improved predictive accuracy in prescribing for patients with nonpsychotic mental health conditions.

PEER is a clinical phenotypic database comprising more than 11,000 baseline EEGs and more than 39,000 outcomes of medication treatment for a variety of mental health diagnoses. Collective findings from 45 studies (3130 patients) provide compelling evidence for QEEG and PEER as a relatively simple, inexpensive predictor of likely patient response to specific antidepressants and likely treatment-related side effects (including suicidal ideation).

Predictive analytics decision-support tools such as PEER help physicians reduce trial and error treatment in mental health and provide more personalized care to patients with depression. Studies show that combining evidence-based practice with objective information facilitates clinical decision making and improves patient outcomes. This can significantly reduce the excess costs – an estimated $8000 per patient per year – for patients who do not receive appropriate medication and are labeled refractory to pharmacologic treatment. There is general agreement that the benefits of predictive analytics include improved patient access to customized care, increased transparency, and accelerated innovation in patient care delivery and services while reducing the total cost of care. No clinical specialty is in greater need of such decision-support tools than behavioral health. By improving the accuracy of prescribing and efficacy of treatment, tools such as PEER may help to improve patient health, provider satisfaction, family and community well-being, and economic outcomes.
